# Instrument–assisted soft tissue mobilization versus myofascial release therapy in treatment of chronic neck pain: a randomized clinical trial

**DOI:** 10.1186/s12891-023-06540-5

**Published:** 2023-06-03

**Authors:** Fatma Shewail, Salwa Abdelmajeed, Mohamed Farouk, Mohamed Abdelmegeed

**Affiliations:** 1 Orthopedic physical therapy department, Faculty of physical therapy, Misr University for Science and Technology, Cairo, Egypt; 2 Orthopedic physical therapy department, Faculty of physical therapy, Cairo University, Cairo, Egypt

**Keywords:** Neck pain, Manual therapy, Physical therapy

## Abstract

**Objective:**

The purpose of this study was to investigate the effect of instrument-assisted soft tissue mobilization (IASTM) versus myofascial release therapy (MRT) on college students with chronic mechanical neck pain (CMNP).

**Methods:**

Thirty-three college students with a mean age of 21.33 ± 0.98 involved in distance learning due to the Corona Virus 2019 (COVID-19) restriction were randomized to receive either IASTM on the upper trapezius and levator scapulae muscles or MRT. Researchers measured their pain with a visual analog scale (VAS), function with neck disability index (NDI), and pain pressure threshold (PPT) with a pressure algometer. The subjects received eight therapy sessions over four weeks and outcome measures were assessed pre and post-intervention. The study was registered as a clinical trial on clinicaltrials.gov (registration number: NCT05213871).

**Result:**

Unpaired t-test showed no statistical significance between the two groups post-intervention regarding improvement in pain, function, and PPT (p > 0.05).

**Conclusion:**

This study showed insignificant differences between groups. However, we did not use a control group, indicating that the improvement in outcomes may not have been caused by the intervention.

**Study design:**

Quasi-experimental two groups pre-posttest clinical trial.

**Level of evidence:**

Therapy, level 2b.

## Introduction

Neck pain is one of the most frequently encountered disorders in clinical settings [1] and is often difficult to diagnose and treat [2, 3]. Neck pain continues to increase in the general population and specific subgroups worldwide [3]. Published data on the prevalence is variable, but it is estimated that 22–70% of the general population will experience pain at some point in life [4–6].

Patients with chronic mechanical neck pain (CMNP) are present with a wide range of symptoms ranging from mild pain and minimal functional limitation to complete disability [7]. Therefore, it has great socio-economic and negative health impacts [1].

Among the identified risk factors for the development of CMNP are a long history of neck pain, worrisome attitude, poor quality of life, and less vitality. The same clinical practice guidelines and its updated revision identified female gender and prior history of neck pain as predisposing factors for the development of a new onset of neck pain. In addition, there is low to moderate evidence that high job demands, history of smoking, low social/work support, and history of low back pain are risk factors for the development of neck pain in general [2, 3].

The international classification of functioning, disability, and health (ICF) endorses functional terminologies in describing health conditions. Therefore, the clinical practice guidelines linked to the ICF classify patients with neck pain into four categories: neck pain with mobility deficits, neck pain with headache, neck pain with movement coordination deficits, and neck pain with radiated upper extremity pain. Each category is presented with clinical findings specific to that category2,3.

There is strong evidence that young individual patients with a duration of symptoms less than 12 weeks can be diagnosed with neck pain and mobility deficits when they are presented with symptoms isolated to the neck and have a limited cervical range of motion (ROM) [2, 8–11]. Moreover, the revised clinical practice guidelines of neck pain [3] identified the patients with a presentation of the following symptoms as having neck pain with mobility deficits: central and/or unilateral neck pain, limitation in cervical ROM with reproduction of familiar symptoms, associated referred shoulder or upper extremity pain.

College students can be a risk population for developing CMNP because of the long hours spent studying in front of computer screens [12]. This can be also triggered by sustained posture and abnormal cervical spine mechanics with tenderness on palpation [1–3]. Additional clinical examination findings of patients with neck pain and mobility deficits include limited cervical ROM, neck pain reproduced at the end of active and passive ROM, restricted cervical and thoracic segmental mobility, associated scapular/thoracic segments pain, and strength deficits in subacute or chronic neck pain [3].

Instrument-assisted soft tissue mobilization (IASTM) has gained wide attention as a relatively new technique in the treatment of muscular tightness and pain. Originally described by Cyriax in 1982, this technique can be performed using different tools. The IASTM uses the same concept by applying an adapted pressure on the tight structures using different-shaped stainless-steel tools with beveled edges to conform to different anatomical structures. Although there is some empirical evidence for its use [14], its effect has not yet been investigated in subjects with CMNP to the authors’ knowledge.

Myofascial treatment is an emerging treatment in different musculoskeletal conditions although its clinical benefits is still not clearly understood [15]. Myofascial release therapy (MRT) aims at restoring the normal length of a tight structure with the target goal of decreasing pain and improving function. Since patients with neck pain are usually presented with myofascial trigger points (MTrPs), MRT can be an effective treatment technique [16].

Since there is a research gap in understanding the effect of IASTM and myofascial MRT in college students with CMNP, the authors of this study were interested in building up evidence for their use. During Coronavirus time, college students were ordered to stay at home to stop spreading infection and as a mitigation strategy. Thus, they had to use the computer for long hours. Therefore, the purpose of this study was to compare the effect of the IASTM technique and MRT on college students studying using distance learning and having CMNP. Researchers hypothesized that there will be no statistically significant difference between the effect of IASTM and MRT on improving pain, function, and/or improving pressure pain threshold.

## Methods

### Study design and setting

This was a prospective quasi-experimental two groups pre-posttest study. Due to the nature of the intervention, we could not blind the participants or investigators to the intervention. The participants, however, were randomly assigned to the two experimental groups. Therefore, we followed a quasi-experimental design. The study was conducted at the Faculty of Physical therapy, Misr University for Science and Technology (MUST). It was approved by the institutional review board (IRB) of the Faculty of Physical Therapy, Cairo University (approval number: PT.REC/012/003381) and was registered on clinicaltrials.gov (registration number: NCT05213871), registration date 28/01/2022.

### Participants

Thirty-three college male and female students were randomized to receive either IASTM on the upper trapezius and levator scapulae muscles (group A) or myofascial release on the same muscles (group B). Both groups received postural correction and strengthening exercises for neck and scapular stabilizers in addition to their assigned treatment. The inclusion criteria were college students between 18 and 25 years old with CMNP localized to the cervical and periscapular regions, who report at least one trigger point in the upper trapezius and/or levator scapulae muscles, and who use the computer daily for at least two hours and are involved in distance learning of at least three months. CMNP was defined as having vague, dull, achy pain in the neck for more than three months with an intensity of at least 30 mm on a 100 mm visual analog scale (VAS) line. Only college students using distant learning during Coronavirus pandemic restriction as stipulated by the school rules were included in the study.

Subjects were excluded if they have any specific neck pathology, radiculopathy, paresthesia, cervical disc pathologies, neurological signs, cervical myelopathy, vertebrobasilar insufficiency (VBI), or acute or subacute neck pain of any nature. Subjects were also excluded if they have any systemic diseases such as rheumatoid arthritis, ankylosing spondylitis, hemorrhage tendency and/or anticoagulation treatment, or spinal instability. Subjects who did not meet the inclusion criteria were also excluded from participation.

### Sample size calculation and reporting of the clinical trial

**Fig. 1:**
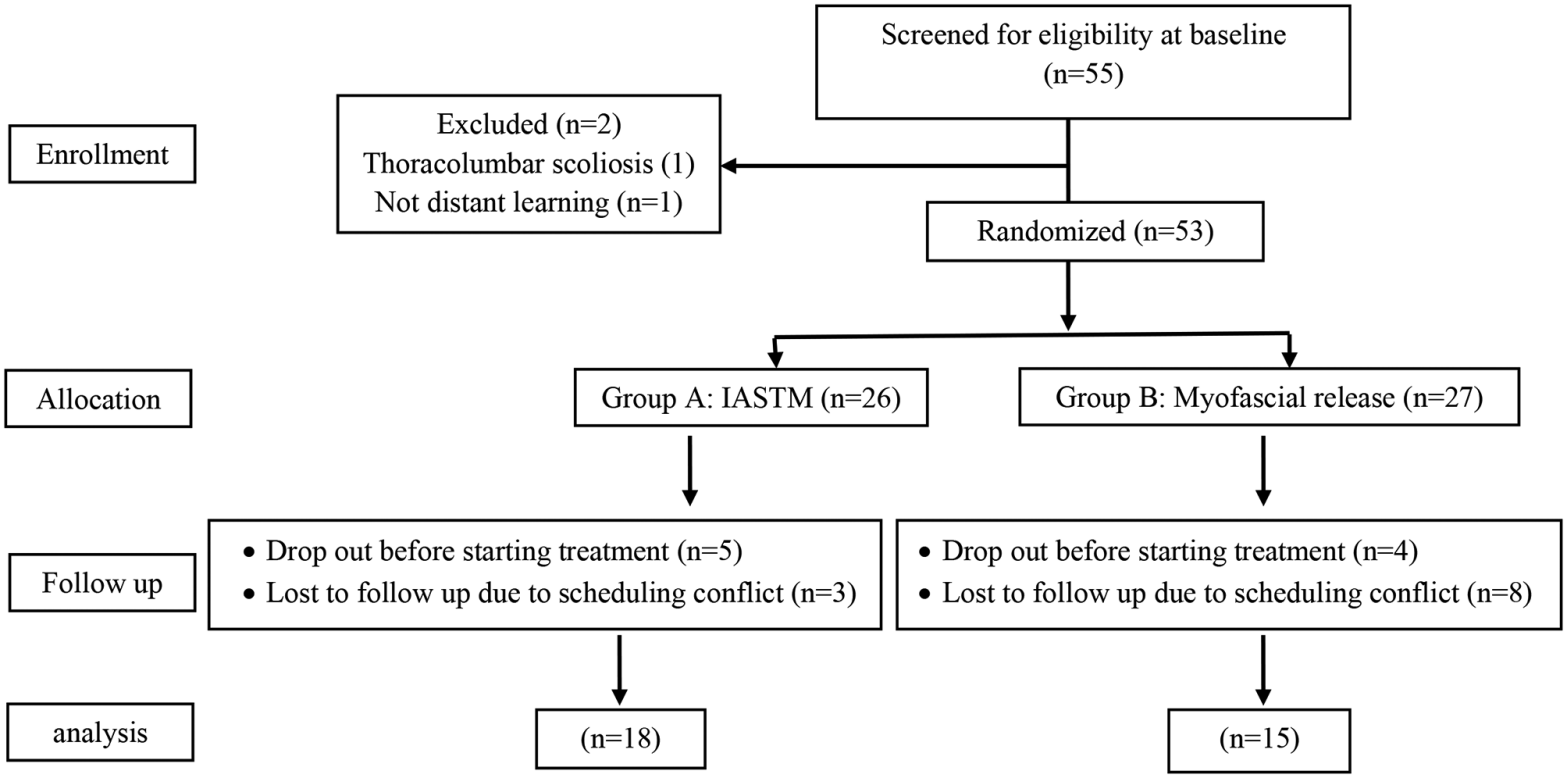
Flow chart outlining the progression of the clinical trial

To detect an effect size of Cohen’s d = 0.80 with 80% power (alpha = 0.05), G*power software (version 3.1.9.7) suggested we need 52 participants (26 in each group) in an independent sample t-test. We ended up, however, with 33 subjects. A flow diagram according to the Consolidated Standards of Reporting Trials (CONSORT) statement is presented in Fig. 1 to illustrate the progression of this clinical trial [17].

### Assessment procedure

After signing the consent form, subjects were screened for eligibility to participate. Their demographic data was then collected. It was important to us after screening for eligibility to apply clearing tests to exclude any red flags. We used the Sharp-Purser [18] and alar ligaments [19, 20] tests for ligamentous hyperlaxity/subluxation/dislocation of the proximal cervical spine. They were also screened for VBI by putting the subjects’ heads in extension, side bending and rotation for 30 sitting from supine and sitting positions and assessed VBI signs and symptoms of dizziness, vertigo, nystagmus, and nausea. The screening was performed for both sides.

When the subject was cleared, he/she was asked to place a mark on the VAS line to indicate the level of pain intensity. A ruler was then used to measure the distance from zero, and the recorded number was rounded to the nearest number. For example, a measure of 5.7 cm was rounded to 6 cm.

Subjects’ functional status was evaluated using the neck disability index (NDI). The NDI is a widely used self-reported outcome measure that assesses functional limitations in patients with neck pain. It has 10 items answered on a 0–5 Likert scale for each item. The total raw score is 50 with higher scores indicating greater disability. Psychometric properties for the NDI are well established in the literature [26–28]. We asked the subjects to choose the answer that best described his/her condition for each item of the NDI. Scores were then tallied, and the total score was calculated.

The subjects were then assessed for the presence or absence of MTrPs. We used previously published criteria for evaluation. This includes the presence of a palpable taut band in a muscle, presence of a hypersensitive point in a taut band, a twitch in a muscle caused by palpation, referred pain produced as a result of compression on a tender point, and/ or presence of classical referred pain pattern. Four out of five findings classified the trigger point as latent, while the five findings classified the trigger point to be active [29].

**Fig. 2:**
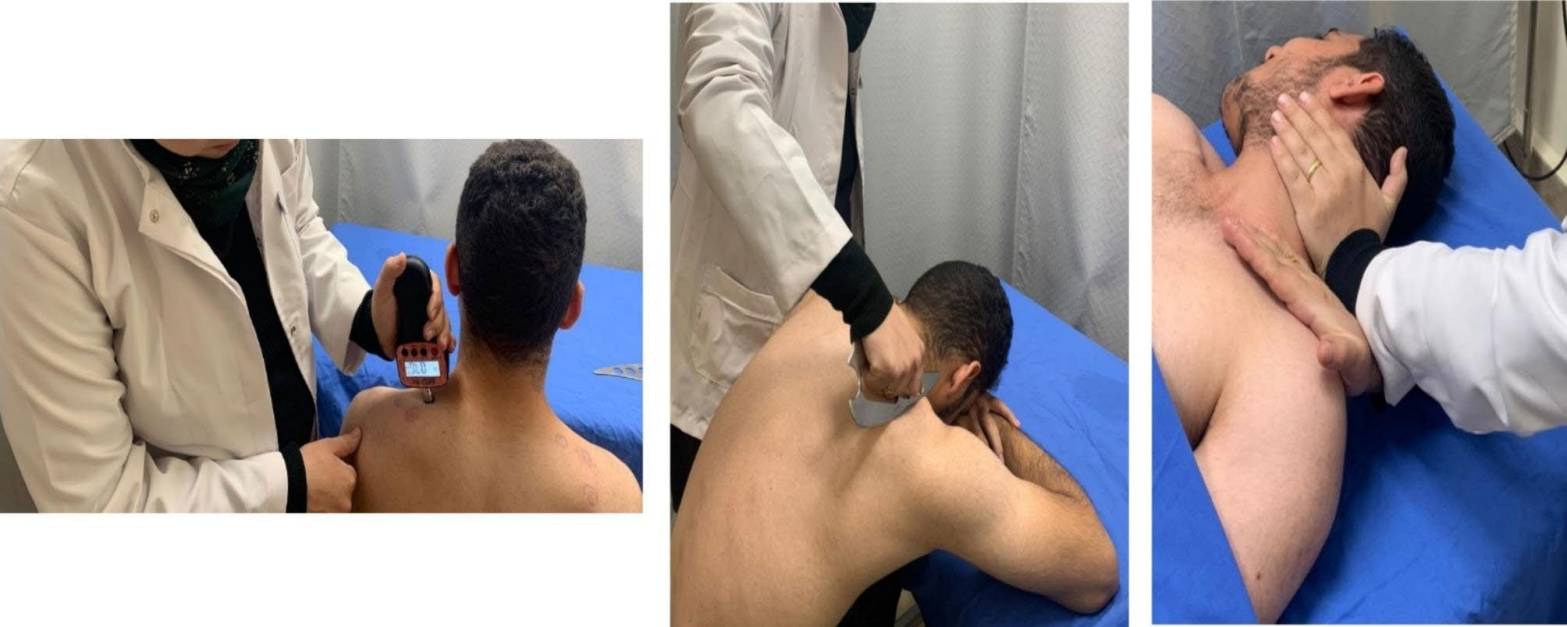
Procedure used in the study. left: assessment of pain pressure threshold, center: application of instrument-assisted soft tissue mobilization (group A), right: manual soft tissue release (group B)

The pressure algometer (model: FPX 50, S/N: 2,010,600,173, JTECH Medical, Midvale, Utah, USA) was then applied perpendicular to the trigger point (Fig. 2). The subject was asked to report when he/she first felt the first discomfort. The compression was then stopped and the value on the algometer screen was recorded. The average of three readings with an interval of 1 min between the trials was recorded [22, 23]. Assessment of pain, function, and pain pressure threshold were performed pre and post-treatment.

### Intervention

Subjects in group A received the IASTM technique using an M2T blade twice a week for four weeks. The subject assumed a comfortable sitting position leaning on a treatment table with the arm crossed to rest the head (Fig. 2). After cleaning the skin of the subject and the blade with alcohol swabs, a lubricant (Vaseline) was applied, and a sweeping technique was used to apply a deep yet comfortable soft tissue mobilization on the upper trapezius from origin to insertion for approximately 3 min. The technique was adjusted if needed to allow the subject to take a break if a sense of burning was felt or if the treatment was uncomfortable. The skin was then cleaned and wiped with tissues. Subjects were instructed that slight hyperemia on the skin is a normal feeling and should subside before the next session [12, 13, 22]. Treatment was applied bilaterally.

Subjects in group B received MRT twice a week for four weeks. While the subject was in a supine position with his/her head supported, the subject’s head was rotated away from the side to be treated, and the therapist crossed her hands as shown in Fig. 2 to take up the slack of the upper trapezius muscle until the tissue barrier was felt. A stretching force was maintained for 30 s at the tissue barrier before moving to a new barrier. The technique was repeated until the end range is reached. Lateral bending of the head was avoided and if more stretching was needed, the therapist depressed the shoulder more at the same time the head was rotated. The subjects were continuously assessed for any discomfort or pain beyond comfortable stretching pain [15, 16]. Both sides of the neck were treated.

In addition, both groups received postural correction and strengthening for neck and scapular stabilizer muscles following the guidelines of Noormohammadpour et al. [31] and Harbut et al. [32] The exercises consisted of active cervical retraction with chin tuck and scapular retraction exercises. Also, manual resistance was applied for cervical lateral bending, extension, and rotation. All these exercises were performed for 3 sets of 10 repetitions with the same frequency of the treatment for each group (twice a week for 4 weeks).

### Data analysis

Data were analyzed using the statistical package for social sciences (SPSS) computer program version 27 software for Windows (IBM SPSS Inc., Chicago, IL, USA). Descriptive statistics were expressed as mean ± standard deviation for continuous variables and frequency distribution (%) for categorical variables. The normality of the data was examined using the Kolmogorov Smirnov statistical test. Comparisons between the two groups were performed using unpaired student t-tests pre and post-intervention for pain, function, and pressure threshold. The alpha level was set at p = 0.05. For the effect size, we used the Cohen’s recommended criteria [24] which is as follows: d ≈ 0.2 indicates a small effect and negligible clinical importance, d ≈ 0.5 indicates a medium effect and moderate clinical importance and d ≈ 0.8 indicates a large effect and high clinical importance.

## Result

**Fig. 3:**
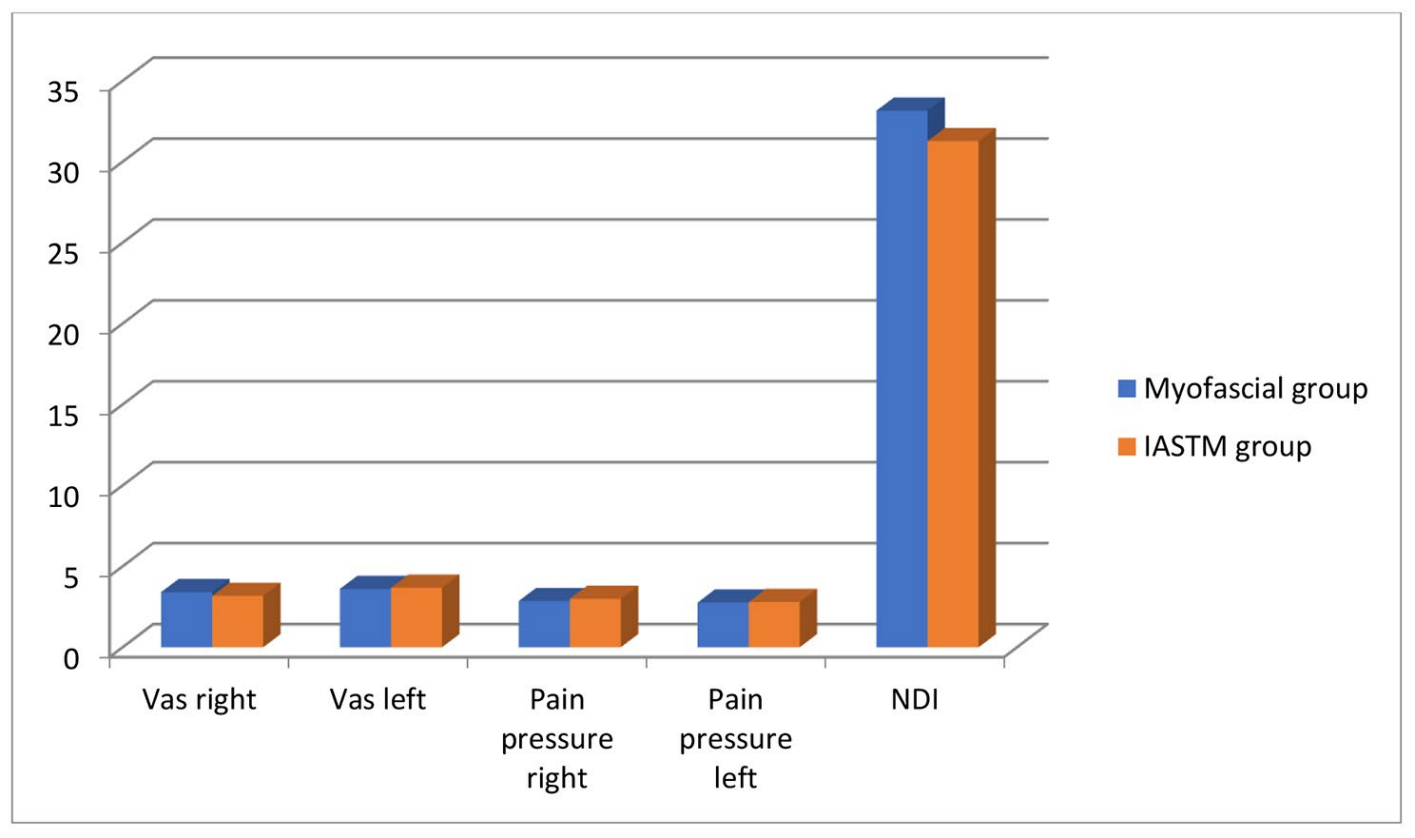
Comparison between the two groups post-treatment

Demographic data are presented in Table 1. There was no significant difference between the two groups regarding subjects’ mean age, body mass, stature, body mass index, or gender (p > 0.05). There was no significant difference between the two groups in all variables measured post-measurement with p > 0.05 as shown in Table 2; Fig. 3.

**Table 1:** Demographic data of the two groups

Data	Myofascial group n = 15		IASTM group n = 18		t-test	p-value
	Mean	SD	Mean	SD		
Age	21.27	1.16	21.39	0.78	0.36	0.72
Body mass	73.40	19.34	66.56	13.23	1.2	0.23
Stature	162.13	4.97	161.82	6.78	0.14	0.88
BMI	27.61	6.99	25.17	4.57	1.2	0.23
Gender	Male 0%, Female 100%		Male 12%, Female 88%		Chi square = 1.7	0.18

**Table 2:** Difference between both groups post-measurements

Data	Myofascial group*	IASTM group*	t-value	p-value	Effect size	MD (95%CI)
VAS right	3.40 ± 1.12	3.17 ± 1.38	0.52	0.6	0.18	0.23 (-0.68-1.14)
VAS left	3.60 ± 1.18	3.67 ± 1.41	0.14	0.88	0.05	-0.07 (-1.02-0.88)
Pain pressure right (kg/cm2)	2.85 ± 0.50	2.99 ± 0.68	0.67	0.5	0.24	-0.14 (-0.57-0.29)
Pain pressure left (kg/cm2)	2.76 ± 0.58	2.79 ± 0.57	0.14	0.88	0.05	-0.03 (-0.45-0.39)
NDI	33.15 ± 5.43	31.27 ± 9.62	0.67	0.5	0.18	1.88 (-3.72-7.48)

*Data are presented as mean ± standard deviation

Abbreviations: IASTM: instrument-assisted soft tissue mobilization, VAS: visual analogue scale, NDI: neck disability index, Right/left: data reported for assessment of each side of the neck, MD: mean difference, CI: confidence interval

## Discussion

The IASTM and MFR techniques appear to have similar effects on pain and disability on college students studying using distance learning and having CMNP. The participants in group A who received IASTM, however, showed a slight clinical improvement in pain, function, and pain pressure threshold. The lack of statistical significance between the two groups may be due to the short duration time of the intervention and/or the lack of a control group. Perhaps this short time was not enough to trigger a statistical significance. It is possible that if the intervention time was more than four weeks, we could have obtained a different result.

The use of valid and reliable outcome measures is always important. In this study, we used VAS, NDI, and pressure algometry to directly address the purpose of the study. It is important to note, however, that the construct of the studied outcome measures is different. In some outcome measures, the lower the scores, the better and it is the other way around in the others. For example, the lower the scores in VAS and NDI, the better the outcome, while the higher scores in the pressure algometry, the better since higher scores indicate higher tolerance for pain pressure.

In this study, our sample included only college students involved in distant learning. During Coronavirus disease (COVID-19), most of the education changed to become online. The world has changed since COVID-19 started and many schools forced students to stay at home and continue their courses online. This has affected students and new complaints such as neck pain emerged due to long hours of sitting in front of computers. We were intrigued to study how interventions like ours could or could not help them. Therefore, we delimited the study participants to college students involved in distant learning.

Although there are similar studies that came to similar results, [21, 22, 25] our study has a population sample with specific characteristics which affected the neck posture because of the “homeschooling” type of learning. In addition, we included a postural correction and strengthening exercise program to augment the program assigned to each group. Although this can limit the generalizability of our result to the general population, we felt it was important to address some of the COVID-19 restriction effects on college students.

While varieties of MFR techniques exist, the type of MFR performed on the neck muscles does not seem to affect the result of pain and pain pressure threshold modulation in subjects with neck pain. In this study, we used gradual stretching for the upper trapezius and levator scapula muscles while in another study, [22] they used a stripping massage applied using the thumb on the upper trapezius muscle. Neither study found a difference between groups in the same outcome measures although there was a significant difference within groups pre and post-treatment. Considering the limitation of both studies, the type of MFR may not be as important as combining more than techniques or boosting the sample size to find a different result.

When compared to conventional physical therapy of therapeutic ultrasound, electrical stimulation, and regular massage, MFR was found to be superior in improving pain, increasing range of motion, and PPT in subjects with subacute and chronic neck pain [16]. Although experimental studies should be conducted to compare intervention with similar constructs, the superiority of MFR intervention in their study may be because manual therapy has superior evidence to passive treatment modalities in patients with neck pain [2, 3]. This was also the case in another similar study [15, 30] although the treatment provided was shorter than ours. Our study and theirs used similar MFR techniques and the three studies showed significant within-group effects from pre- to post-treatment.

This study can be viewed within the context of several limitations. First, the sample size was small, and we had a large dropout rate, either due to COVID-19 restrictions, scheduling conflicts, and/or ineligibility criteria to participate. We could have obtained a different result if we had a larger sample size. The small sample size also limited the generalizability of the result which affects the external validity of the study. Another factor that may limit the generalizability of the results is the fact that the age range and hence the characteristics of participants are small to extrapolate the findings to the general population. Second, our intervention was for a short time. Again, we could have obtained a different result with a long time or with more visits. In addition, we did not follow up with participants beyond four weeks and we could not infer the long-term effect of our intervention.

Third, we assessed trigger points found only in two muscles: the upper trapezius and levator scapulae. Different muscles have different referral patterns and could have been important to study in addition to these two muscles. Fourth, we did not have a control group to better investigate the effect of our intervention, this limits the result of this study. We recommend that future studies include more muscles in their assessment and intervention, apply the same intervention on larger sample size, increase the intervention time by more than 4 weeks, have a long-term follow-up of participants, add a control group, and study other instrument-assisted techniques on subjects with CMNP.

It is important to mention that we did not conduct this study on COVID patients. It was conducted during COVID time though. Being infected with COVID was not a criterion of inclusion or exclusion, rather, it was a factor for which our participants were restricted from engagement in regular college life (probably like the rest of the world). We might though have a subject or two who was considered a “long hauler” but unfortunately, we do not have documentation of this.

## Conclusion

This study showed insignificant differences between groups. However, we did not use a control group, indicating that the improvement in outcomes may not have been caused by the intervention.

## Data Availability

The datasets used and/or analyzed during the current study are available from the corresponding author on a reasonable request.
